# Hybrid Deep Neural Network for Handling Data Imbalance in Precursor MicroRNA

**DOI:** 10.3389/fpubh.2021.821410

**Published:** 2021-12-23

**Authors:** Elakkiya R., Deepak Kumar Jain, Ketan Kotecha, Sharnil Pandya, Sai Siddhartha Reddy, Rajalakshmi E., Vijayakumar Varadarajan, Aniket Mahanti, Subramaniyaswamy V

**Affiliations:** ^1^School of Computing, SASTRA Deemed University, Thanjavur, India; ^2^College of Automation, Chongqing University of Posts and Telecommunications, Chongqing, China; ^3^Symbiosis Centre for Applied Artificial Intelligence, Symbiosis International (Deemed University), Pune, India; ^4^Symbiosis Institute of Technology, Symbiosis International (Deemed University), Pune, India; ^5^School of Computer Science and Engineering, University of New South Wales, Sydney, NSW, Australia; ^6^The University of Auckland, Auckland, New Zealand

**Keywords:** bioinformatics, precursor microRNA, hybrid deep neural network, deep artificial neural network, deep decision tree classifier

## Abstract

Over the last decade, the field of bioinformatics has been increasing rapidly. Robust bioinformatics tools are going to play a vital role in future progress. Scientists working in the field of bioinformatics conduct a large number of researches to extract knowledge from the biological data available. Several bioinformatics issues have evolved as a result of the creation of massive amounts of unbalanced data. The classification of precursor microRNA (pre miRNA) from the imbalanced RNA genome data is one such problem. The examinations proved that pre miRNAs (precursor microRNAs) could serve as oncogene or tumor suppressors in various cancer types. This paper introduces a Hybrid Deep Neural Network framework (H-DNN) for the classification of pre miRNA in imbalanced data. The proposed H-DNN framework is an integration of Deep Artificial Neural Networks (Deep ANN) and Deep Decision Tree Classifiers. The Deep ANN in the proposed H-DNN helps to extract the meaningful features and the Deep Decision Tree Classifier helps to classify the pre miRNA accurately. Experimentation of H-DNN was done with genomes of animals, plants, humans, and Arabidopsis with an imbalance ratio up to 1:5000 and virus with a ratio of 1:400. Experimental results showed an accuracy of more than 99% in all the cases and the time complexity of the proposed H-DNN is also very less when compared with the other existing approaches.

## Introduction

The problem of imbalanced data has often been recognized as a significant challenge in Machine and Deep Learning. The ML classifiers tend to get influenced by the majority classes when it comes to imbalanced data. This results in a poor classification of the minority classes. Whenever the class sizes are significantly different, the classification methods perform much better in the larger class, achieving accurate results, whereas the minority class seems to have a very poor recall. The classification of one class being considerably neglected in comparison to another is still one of the most difficult problems to solve in the creation of new classification models today. Bioinformatics is one of the major streams that provide a large number of highly imbalanced data. These enormous datasets require a great solution that can handle such problems and are appropriate for such imbalance ratios. One of the major data imbalance problems in bioinformatics is the identification of the precursor micro RNAs (pre-miRNA) from the genome. The miRNA is a special type of non-coding RNA. They have a length of 21 nucleotides and they play a key role in the posttranscriptional regulation of gene expression. The miRNA is a minute, endogenous RNA having a hairpin-like structure. Numerous key biological processes, such as disease genesis and progression, are regulated by miRNAs. They can act as an oncogene or tumor suppressor in a variety of cancers assisting in prognosis prediction, therapeutic assessment, and disease diagnosis (1). A promising therapy for breast cancer is delivered by the latest miRNA-based drugs (2).

The genome data consists of hundreds of thousands of sequences out of which there will be only a few miRNAs. Moreover, there are several other similar RNAs which makes it even more difficult to differentiate them. Further, the minority class usually contains a small number of high-variability instances, making it more challenging for a classifier the generalization of the new dataset. So, to find them in the highly imbalanced genome data and classify them is a great challenge and hence there is a need to find an optimal solution for identifying them with a simple procedure. Although the computational methods have been necessary tools in miRNA gene finding and functional studies (3). The Machine Learning (ML) techniques have made it possible to classify them to a good extent (4). Machine Learning and Deep Learning (DL) have taken control over every field and are providing adequate solutions for every complex problem. There are few algorithms proposed for which the True Positive Rate (TPR) is very less and complexity is more. For the genome datasets, there are many negative samples and very few positive samples of miRNA sequences. Generally, this type of problem is related to Imbalanced data. To handle such Imbalanced data there are a few solutions out of which Sampling is the most famous and efficient way. There are two variants of sampling: Oversampling and Undersampling. Oversampling is the technique in which the fewer samples in the dataset will be upscale (in other words upscaling the minorities) to make the data to be balanced. In this technique, many dummies for fewer samples are generated. When the dataset is with a smaller number of samples (both positive and negative) this technique will help to build a model with an adequate number of samples. Under Sampling is the technique in which the greater number of samples in the dataset will be downscaled (in other words downscaling the majorities) to make the data to be balanced. In this technique, the Selection method or Random sample method is chosen. In general, when the dataset is very large, the model will take a huge time to execute, this technique will help us to overcome this issue. In one of the proposed models (5), they chose an Imbalance Ratio (IR) up to 1:2000 (meaning one positive sample and 2,000 negative samples), and different ratios like 1:1, 1:100, 1:500, 1:1000, 1:1500, 1:2000. To improve the model and follow the consistency, different IRs like 1:2500, 1:3000, 1:3500, 1:4000, 1:4500, 1:5000 have been added. A model was proposed where, in addition to the Animal and Plant dataset, the Human and Arabidopsis dataset with IR up to 1:5000 and virus dataset with IR up to 1:400 was considered to enhance the performance (6).

The existing systems like Multi-layer perceptron (MLP), Support Vector Machine (SVM), Deep Self-Organizing Map (DeepSOM), Deep Elastic SOM(DeSOM), Deep Elastic Ensemble SOM (DeeSOM), Deep Belief Neural Network (DeepBN) can solve this problem to some extent only. The Deep Architectures which work without SMOTE (Synthetic Minority Oversampling Technique) (7) take more time and accuracy is also poor, the other systems stated above also produce low accuracy if the class imbalance ratio is gradually increased. Most of the existing works so far have not considered multiclass pre-miRNA classification having an imbalanced dataset, even if they have discussed their performance is not up to the mark and hence end up having low accuracy. The accuracy of the existing work also goes down as the imbalance ratio is increased as it is given in the real-time scenarios. So, to address the problems like high run time and low accuracy faced in these existing systems, we propose a Hybrid Deep Neural Network (H-DNN) architecture, which is implemented by integrating the Deep Artificial Neural Networks (ANN) with a Deep Decision Tree classifier (Deep DT). Deep ANN is capable of optimizing a wide range of coefficients. As a result, Deep ANN can tolerate a much larger variation than traditional models. With enough training, Deep ANN will be prepared for any circumstance. It is a type of machine learning technology with a large memory capacity since it is capable of remembering every call. On imbalanced data, DT usually performs effectively. They operate by understanding a hierarchy of if/else scenarios, which forces them to address both majorities as well as minority classes. The main contributions of the proposed work can be listed as follows:

- Preprocessing of multiclass genome dataset and selection of features using Select-K-Best and Recursive Feature Elimination.
- Building proposed H-DNN framework by integrating Deep Artificial Neural Network and Deep Decision Tree for classification of miRNA from a multiclass genome dataset.
- Apply the H-DNN architecture with different imbalance ratios of multiclass genome dataset consisting of Animal, Plant, Arabidopsis, Human, and Virus genomes.

The rest of the paper is organized as follows: Section Related Work discusses the related work. Section Proposed Work discusses the proposed H-DNN architecture and its components. Section Experimental Work details the experiments and presents the results of H-DNN and section Results and Discussions summarizes the proposed approach and its future enhancements.

## Related Work

Due to the imbalanced class distributions in the data, handling such class imbalance problems and drawing a large amount of interest toward it, has become the major issue. To deal with these class imbalance problems many ensemble methods are proposed but most of them focus on the two-class imbalance problems, leaving many issues unsolved in the multiclass imbalance problems. To solve those multi-class problems efficiently an algorithm called AdaBoost.NC was proposed. It produced better results when compared with other popular ensemble Techniques (8). As the availability is expanding in all kinds of fields it becomes difficult to perform analysis and progress from raw data to decision-making processes. Even though new data engineering techniques are showing great success in solving real-world problems, learning from imbalanced data has emerged as a new challenge. To enable efficient learning from imbalanced data it is required to have new approaches, understandings, algorithms, principles, and tools, because of its complex characteristics (9, 10). Class imbalances make supervised learning problems difficult because some classes have more examples than others. The existing methods have completely focused on binary-class cases. A dynamic sampling method for Multilayer Perceptron (MLP) was proposed to solve the multi-class imbalance problems. To train the MLP, DyS dynamically selects informative data. This Dynamic sampling technique has performed well when compared with other techniques (11). There are several other preprocessing techniques for the data, classification algorithms, and evaluating the model involved in modeling methods (12). The important regulators of gene expression are MicroRNAs (miRNAs). The genetic loss of the tumor suppressor miRNAs or Amplification and the overexpression of one's 'oncomiRs' is related to human cancer and is enough to cause tumorigenesis in mouse models. Besides, the depletion of global miRNA by genetics and in the components of miRNA biogenesis machinery epigenetic alterations is oncogenic. All these together with novel miRNA regulatory pathways and factors show how important miRNA dysregulation is in cancer (13). Even after identifying hundreds of miRNAs in different species, a lot of others remain unknown. So, in understanding miRNA-mediated posttranscriptional regulation mechanisms the discovery of new miRNA genes is an important step. The biological approaches in identifying miRNA genes might be limited. So, to overcome those limitations in the biological approaches sophisticated computational approaches are followed to identify the possible miRNA (14). Some of the work deals with the miRNAs in terms of diagnosing dreadful diseases in the medical field. Several web-based bioinformatics tools were proposed for the analysis of miRNA such as expression, detection, etc. with an easy-to-use model that doesn't require any previous knowledge of utilization (15). A Support Vector Machine (SVM) based approach was proposed to classify the real and pseudo miRNA. It achieved an accuracy of up to 90% (16). The model was exclusively for the human dataset but the remaining datasets could also be used. In this work, then genomic information was not required. Later, software that classified the RNA of mammals, nematodes, and also urochordates was proposed. A new methodology was proposed based on Support Vector Machine (SVM) and named as Microprocessor SVM, which predicted with 50% accuracy (17). To improve the accuracy of this model, the model was trained with another classifier and ensemble both to get better results than acquire 90% in total. Another work dealt with the miRNA sequences of the mouse and human using the SVM approach (18). The model worked with around 377 mouse data and 475 human data and with the candidates, the dataset had 3,441 humans and 3,476 mouse samples in total. The classification was done according to the structure of the miRNAs, the model names, and the mirCoS. One of the works also dealt with the use of ab initio Prediction model clustered microRNA identification (19).

An application for the classification of pre-miRNAs was proposed which dealt with the classification of the mammalian miRNAs such as Human, Rat, and Mouse (20). This approach analyzed the genome properly using the model named ab initio which gives an accuracy of up to 60% when they undergo cross-validation. To analyze the structure of miRNA, a tool named MiRFinder was proposed which was built with the SVM model (21). The structure of the miRNA is a hairpin-like structure, the prediction mainly focused on the structure and later the features were used to predict the miRNA. This tool was mainly used for the genome-wide search of the miRNAs. The dataset which has 697 real miRNA and 5,428 pseudos from different sources was collected and experimented with. The tool gained sensitivity up to 93%. Another model named miRenSVM was proposed that gave good results for the data in miRBase 13.0 animal and other species (22). For animal data in miRBase 13.0, it gave 100% sensitivity. A novel RNA effective classification methodology namely microPred was proposed which classified the types of miRNA using the Machine Learning approach (23). It used a classifier namely microPred, which also could handle the data imbalance issue as well as new feature extraction relevant biologically. It achieved a specificity of 97.28% and a sensitivity of 90.02%. The model gave the prediction rate of 92.71% for humans and 94.24% for the virus. Another new model was developed that used a method named YamiPred, which is an ensemble of Genetic Algorithms (GA) and Support Vector Machine (SVM) for the prediction of miRNAs (24). The model mainly used a human dataset to predict the human miRNAs. The model appeared to be robust and efficient with different performance characteristics. Table 1 illustrates the comparison of various state-of-the-art methodologies used for the classification of miRNA.

**Table 1:** Comparison of various state-of-the-art methodologies.

**Author**	**Methodology used**	**Metrics**	**Dataset**
Park (25)	Recurrent Neural Network (RNN).	Various Metrics were used. Experimental results yielded an F1-score of 0.93 for the Human dataset, 0.94 for the Cross-Species dataset, and 0.93 for the New pre-miRNA dataset.	Human, Cross-Species and New miRNA
Thomas et al. (26)	Deep Learning with a restricted Boltzmann machine (RBM) for classification and Modified sampling technique is applied to address the class imbalance problem.	Various Metrics were used. The model gave an accuracy of 0.968	Human Dataset
Stegmayer et al. (27)	Deep Self-organizing maps (DeepSOM) with Clustering	Various evaluation metrics were used. The model provided almost 95% of Gm and 97% of accuracy for the most imbalanced data.	H. sapien and A. thaliana, Animal, and Plant
Bugnon et al. (5)	Hybrid deep learning method with CNN and LSTM.	Various Metrics were used for different imbalance ratios. Experimental results yielded an F1-score of more than 40% for Animal and more than 33% for the Plant dataset.	Animal and Plant
Tang et al. (28)	CNN with different types of feature learning and encoding methods.	The results gave an accuracy of 99.25% and an F1-score of 99.25% for Rfam-300	Rfam-300, Rfam-120, Rfam-60, Rfam-30
Shi et al. (29)	Localized multiple kernel learning model with a nonlinear synthetic kernel (LMKL-D)	The model gave an accuracy of 98.3%, sensitivity of 93.06 Specificity of 99.27 and mean of 96.11.	pre-microRNAsand pseudo pre-microRNA
Yones et al. (30)	Convolutional deep residual neural network	The model provided a precision of 0.93 for A. thaliana full genome, 0.67 for A. gambiae, and 0.50 for H. Sapiens	A. thaliana, C. elegans, A. gambiae, and H. Sapiens
Proposed HDNN Methodology	Artificial Neural Network embedded with Decision Tree	Various metrics were used for evaluation for different imbalance ratios. The results yielded F1 Score of more than 0.50 (0.50–0.93) for Animal, more than 0.58 (0.58–0.95) for Plant, more than 0.60 (0.60–0.94) for Human, more than 0.50 (0.50–0.95) for Arabidopsis and more than 0.50 (0.50–0.94) for Virus.	Animal, Plant, Human, Arabidopsis, and Virus.

## Proposed Work

In this section, we discuss the proposed Hybrid Deep Neural Network approach for the classification of miRNA from genome data. Different genome datasets have been considered in our proposed work such as Animal, Plant, Human, Arabidopsis, and Virus datasets. First, the dataset is preprocessed and all the important features are selected and extracted and the non-useful features are omitted. For feature selection and extraction feature selection Algorithms like Select-K-Best and Recursive Feature Elimination are used. After preprocessing the dataset, the proposed H-DNN algorithm is implemented. The proposed H-DNN framework is the integration of Deep ANN with the Deep DT. Then the implemented model has experimented with the different genome datasets (31).

### Preprocessing

**Figure 1:**
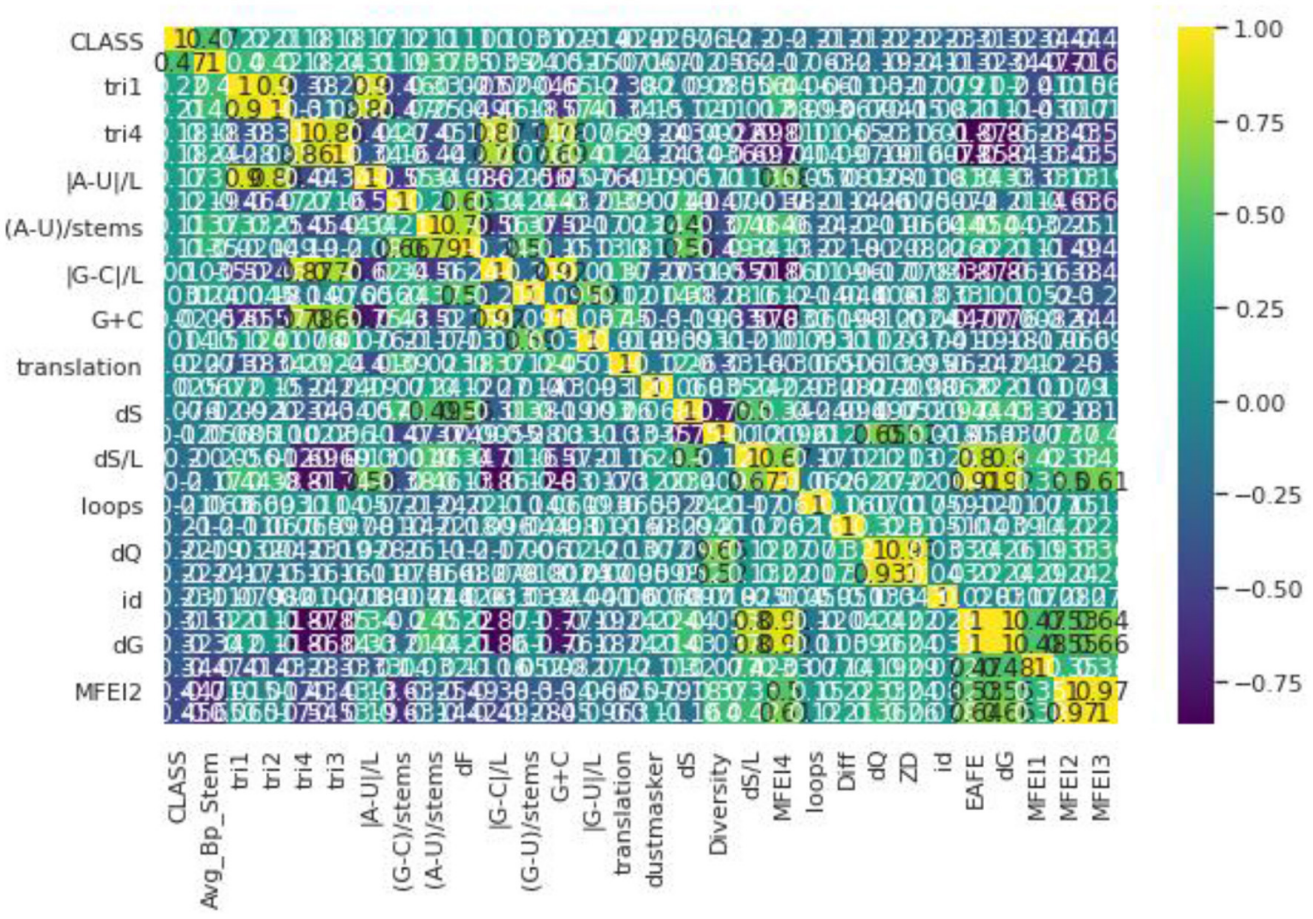
Correlation matrix.

Pre-processing refers to Feature Selection or Feature Extraction. Different Feature Selection algorithms have been applied with the dataset (32). To have a clear idea of feature selection, the dataset has to be analyzed first. Different genome datasets have been considered in our proposed work such as Animal, Plant, Human, Arabidopsis, and Virus datasets. Even though these datasets have a different number of samples in each, they have the same number of features (33). The dataset used here has a total of 30 features in which one is a serial number, hence we omit that attribute. The remaining 29 features are taken into consideration and passed to the Feature Selection Algorithms (34). Figure 1 illustrates the correlation matrix plotted for all the features and a heat map is produced out of that as shown. The correlation matrix helps us to characterize and sum up the data for our understanding. Correlation, here, helps us to compute the association between two variables by testing the effectiveness of their linear relationship. After estimating the correlation matrix, the Feature selection Algorithms like Select-K-Best and Recursive Feature Elimination are used. In the Select-K-Best algorithm, the features are assigned with particular scores. The Selection is generally made based on the scores which are greater than the mean of all the scores in this Feature Selection (35). In the Recursive Feature Elimination, the rank is assigned to each feature and the ranks may be the same for more than one feature. The feature selection is made based on the ranks that are assigned. Generally, the selection is done based on the first few ranks like 10, 12, etc., based on several features (36). The Recursive Feature Elimination with Cross-Validation is used. This gives scores between 0 and 1. The 0, being the least important and one is the most important feature to build any model. This algorithm is applied to the dataset and the results state that all the features are nearer to the one (37). Hence, all the features are important. This proves that there is no need for removal or elimination of any feature from the data so all 29 features are used to build the proposed model (38).

### Proposed H-DNN Architecture

In this section, we discuss our proposed work. We have developed a Hybrid Deep Neural Network (H-DNN) wherein we have integrated Deep Artificial Neural Network and Deep Decision Tree (DT) classification technique for classifying the miRNAs in the genome data. The integration of feature extraction using machine learning techniques and feature analysis and classification using Deep Neural Network has provided better results. The experimental results have been carried out using Animal, Plant, Human, Arabidopsis and, Virus genome datasets. The results are then analyzed and compared for the model using Deep ANN and Deep DT. Deep ANN here helps in analyzing the features by optimizing a wide range of coefficients. As a result, Deep ANN can tolerate a much larger variation than traditional models. Deep ANN gets well prepared for any circumstance with sufficient training. It is a type of machine learning technology with a large memory capacity since it is capable of remembering every call. DT usually performs effectively in case of imbalanced data by understanding a hierarchy of making decisions for different scenarios, which forces them to address both majorities as well as minority classes.

The structure of ANN consists of a neuron, a propagation function, and a bias. Consider a neuron *k* having an input *I*_*k*_(*t*) received from the previously connected neurons. The neuron will have an activation function *a*_*k*_(*t*), an optional threshold θ_*k*_, an activation function *f* for computing new activation for time *t* + 1 as given in Equation 1, and a function *f*_*out*_ for computing the activation output which is formulated as given in Equation 2.


(1) $$\begin{array}{l} a_{k}\left(t+1\right)=f\left(a_{k}\left(t\right),\;p_{k}\left(t\right),\theta_{k}\right) \end{array}$$

(2) $$\begin{array}{l} O_{k}=f_{out}(a_{k}\left(t\right) \end{array}$$

The input, *I*_*m*_(*t*) to the neuron *m* from the output of *k*, is computed using the propagation function defined as given by Equation 3. After adding the bias, *w*_0*m*_, we formulate the input as given in Equation 4.


(3) $$\begin{array}{l} I_{m}\left(t\right)=\underset{k}{\sum }O_{k}\left(t\right)w_{km} \end{array}$$

(4) $$\begin{array}{l} I_{m}\left(t\right)=\underset{k}{\sum }O_{k}\left(t\right)w_{km}+w_{0m} \end{array}$$

Artificial Neural Networks (ANNs) have several different coefficients, which the ANNs can optimize. So, when compared with the traditional models, ANNs can handle much more variability (39). This makes ANN a stronger model when it comes to memorization (40). A decision tree is a supervised learning Algorithm. Both regression and classification problems can be solved using Decision Trees. In the tree representation, each leaf node represents a class label and the internal node of the tree represents the attributes. Decision trees include three main units that are node, branch, and leaf. The node is the decision. The branch is the potential decision, and the leaf is the potential outcome of each decision. The DT illustrates the long-term consequences of certain decisions. The trade-offs and probabilities can also be expressed using decision trees. In a decision tree, after every level, we have a split taking place which in turn results in the next level. Therefore, the major challenge in Decision trees is to identify the attribute for the root node in each level. This is called attribute selection. There are various popular attribute selection measures in decision trees we have used Gini Index for attribute selection in our framework. Gini index can be formulated as shown in Equation 5.


(5) $$\begin{array}{l} Gini\;Index\;=\;1-\overset{\;}{\underset{j}{\sum }}p_{j}^{2} \end{array}$$

here *p*_*j*_ denoted probability for class *j*. Gini index is used here as a cost function for the split evaluation in feature selection. When compared with other algorithms, the decision tree needs less effort for data preparation during preprocessing. It does not require scaling of data or normalization of the data. In addition to this transparency and ease of use makes the decision tree a better model to handle large amounts of data.

**Figure 2:**
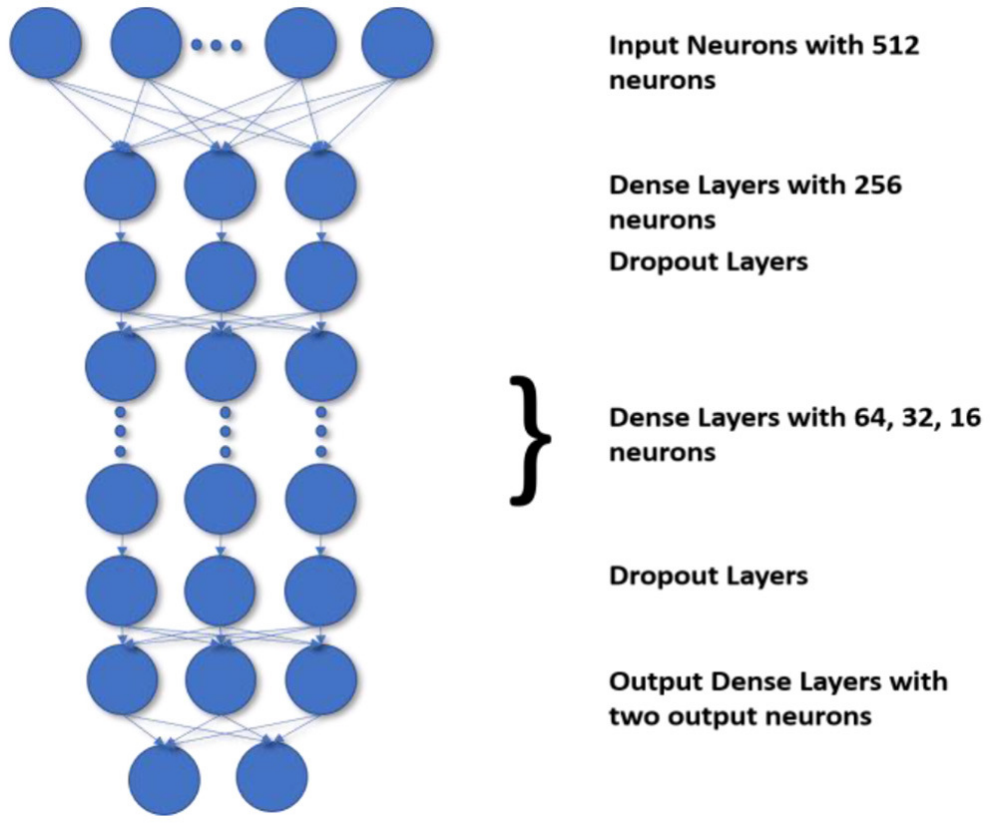
Structure of ANN in each node of Decision Tree in the proposed framework.

The most challenging task is to handle the imbalanced data. The current state-of-the-art methods do not define the decision process take more time and accuracy is also poor. Generally, the state-of-the-art frameworks produce low accuracy if the class imbalance ratio is gradually increased. Before DNN, decision trees were the standard method used to improve accuracy and performance. Though as compared to DNN the accuracy gained by using a decision tree for classification are less the DT preserves the interpretability. So to preserve the DT's interpretability and match the DNN's performance, we use the H-DNN architecture wherein we integrate the Decision Tree and the ANN. The H-DNN model consists of a DT wherein each node of the DT will have an ANN model. The DT is used to make the classification of miRNA in an H-DNN, maintaining high-level interpretability. Every node in a decision tree, on the other hand, is a neural network that makes low-level decisions. First, a hierarchy for DT is constructed and then the sample is passed through the ANN, which is a fully connected neural network, in each decision node. Then the model computes the analysis by executing the final fully connected layer as a series of integrated decision rules. The final prediction is a culmination of these decisions. The ANN structure proposed in this paper deals with a dense layer as input layer with 512 nodes and ReLu activation function followed by another dense layer with 256 nodes and a dropout layer, then three more dense layers with 64, 32, 16 nodes respectively with ReLu activation function and a dropout layer and finally one dense layer with two nodes as output layer. This ANN structure, as illustrated in Figure 2, is embedded into each node of the decision tree for making the decision rules. In the fully connected layer of each node of the decision tree, the inner product of the matrix-vector is calculated and an inner product that constitutes the maximum value is chosen for the class decision rule as shown in Equation 6.


(6) $$\begin{array}{r} \left[w_{1}\;w_{2}\;\vdots \;w_{n}\;\right]\left[\vdots \;x\;\vdots \;\right]=\left[\left(x,w_{1}\right)\;\left(x,w_{2}\right)\;\vdots \;\left(x,w_{n}\right)\;\right]\\ =\left[y_{1}\;y_{2}\;\vdots \;y_{n}\;\right]\to argmax\left(y\right) \end{array}$$

Algorithm 1 illustrates the H-DNN algorithm that uses the Deep Decision Tree having Artificial Neural Network embedded in each root node. The ANN framework in the proposed model has a single input layer, a few hidden layers, and a single output layer. In the final layer of the hidden layer, the decision rule for splitting the tree has been incorporated using Gini Index. Line 1 and 2 in Algorithm 1 initialize the tree and the minimum criteria to be considered for making the decision rule. The Deep ANN is embedded into each node of the Deep DT as illustrated from lines 3 to 10. For a given input node and input, line 4 and 5 illustrates the implementation of the first layer, i.e., the input layer of the Deep ANN. Using the output of the input layer the hidden layer is calculated using lines 6 and 7. In line 8 the Gini Index is calculated to determine the decision rule for splitting the node or tree further. Using the output of the hidden layers the output layer is constructed in lines 9 and 10. In line 11 the Backpropagation algorithm is called to adjust the weight according to the errors. In lines 12 to 15, the decision rule has been implemented. Once the decision rule is applied the nodes are split accordingly as shown in line 16. This process is then implemented for each node until all the nodes are processed and no further nodes can be split.

**Algorithm 1:** Proposed H-DNN.

***Input:*** Genome data, *D*, with attributes, *a*
***Output:*** Decision Tree with classes
1. *tree* = { }
2. *minVal* = 0
3. **for** *each a ϵ D* **do**://Considering Input Layer of ANN
4. **for** *each node n* ∈ *a and input x*_*n*_ **do:**
5. *out*_*n*_ = *x*_*n*_//Considering Hidden Layer of ANN
6. **for** *each hidden node h* **do:**
7. $out_{h}=\underset{n}{\sum }\emptyset \left(w_{hn}.out_{n}\right)$
8. *g* = *GiniIndex*(*out*_*h*_, *d*)
9. **for** *each output node k* **do:**
10. $out_{k}=\underset{k}{\sum }\emptyset \left(w_{kh}.out_{h}\right)$
11. *Train ANN using Backpropagation(out*_*h*_*)*
12. **if** *g* < *min Val* **then**
13. *minVal* = *g*
14. *tree*′ = {*a*}
15. *partition*(*tree, tree*′)
16. ***repeat*** till all the partitions are processed
17. ***return*** tree

Algorithm 2 illustrates the implementation of the backpropagation algorithm used for generalizing the Neural Network (NN) by fine-tuning the weights based on the previous iteration's error rate. After applying the forward propagation, the backpropagation algorithm is called to fine-tune the NN. From lines 1 to 6 in Algorithm 2 all the required parameters are initialized. From lines 7 to 13 the backpropagation process is applied to the neural network.

**Algorithm 2:** Modified Backpropagation(a).

1. *d* ← *training dataset mxn dimension*
2. *y* ← *class labels*
3. *w* ← *weights*
4. *l* ← *number of layers* 1, ….., *z*
5. $error_{ij}^{l}\leftarrow error\;for\;each\;i,\;j,\;l$
6. $b_{ij}^{l}\leftarrow 0\;for\;each\;i,\;j,\;l$
7. ***for*** *i* = 1 *to m* **do:**
8. *d*^*l*^ ← *a*(*z*) − *y*(*i*)
9. $b_{ij}^{l}\leftarrow b_{ij}^{l}+a_{j}^{l}.b_{i}^{l+1}$
10. ***if*** *j* ≠ 0 **then**
11. $error_{ij}^{l}\leftarrow \frac{1}{m}b_{ij}^{l}+\lambda w_{ij}^{l}$
12. ***else***
13. $error_{ij}^{l}\leftarrow \frac{1}{m}b_{ij}^{l}$

**Figure 3:**
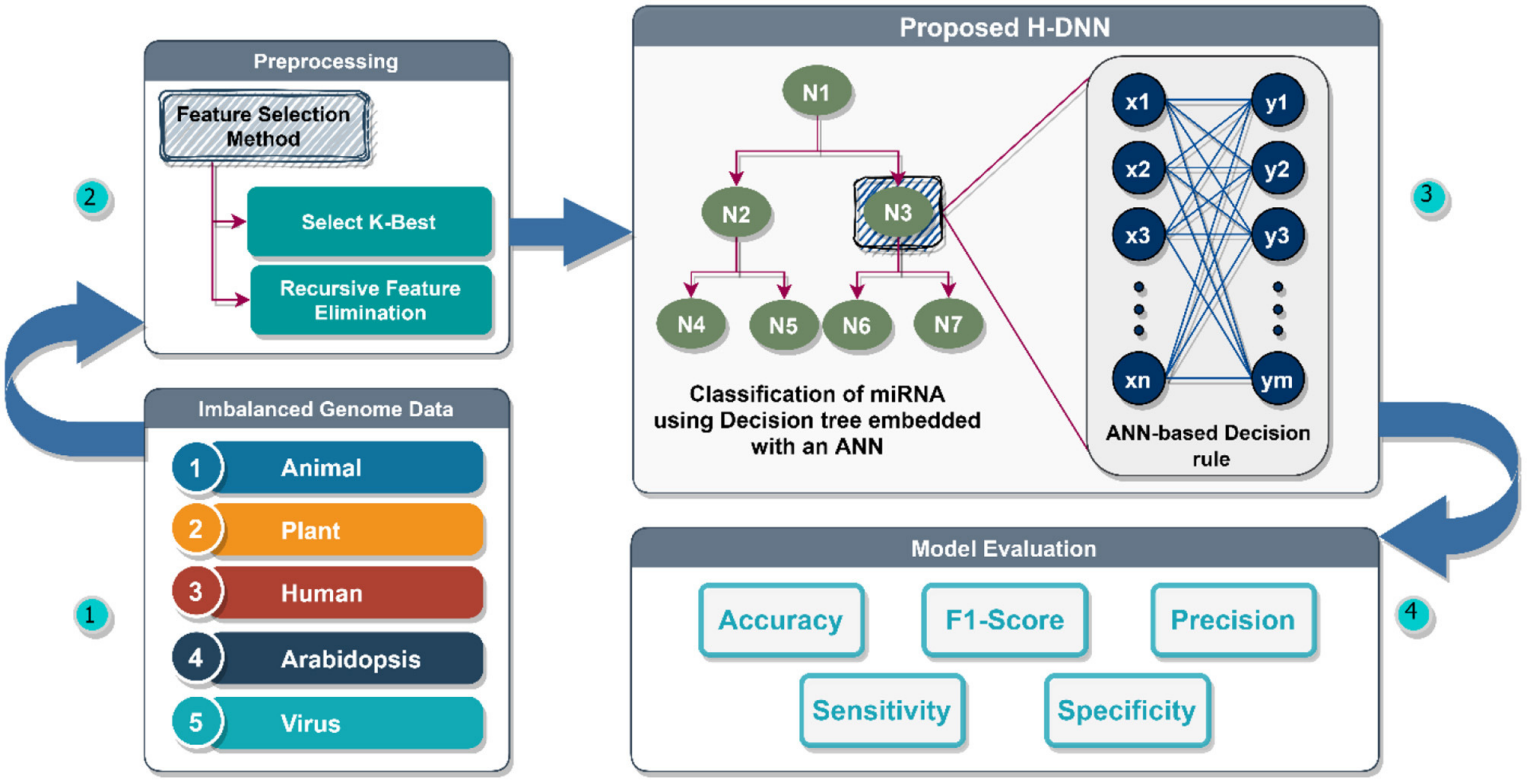
Proposed H-DNN framework.

The proposed architecture can be divided into four modules as illustrated in Figure 3. Module one is the Imbalanced genome dataset which has the collection of Plant, Animal, Human, Arabidopsis, and Virus genome data. This data is preprocessed in module two wherein the feature variables are correlated to find the relationship between the features. In this module, the required features are extracted using the Select-K-Best Method and Recursive Feature Elimination Method, and the rest of the unwanted attributes are discarded. After the feature selection and extraction, the preprocessed data is passed to the third module. The third module is the proposed H-DNN architecture. The H-DNN architecture is the integration of the Decision Tree and the ANN framework. The ANN framework used here helps to avoid overfitting and thereby increasing the performance. The Decision tree helps to make a decision and examine the benefits and cons of each final option. Every node in a decision tree, on the other hand, is a neural network that makes low-level decisions. The inputs of each node of the Decision Tree are passed to ANN for making the decision and analyzing the probability values of the class. The final evaluation of the model is done in the fourth Module, the Model Evaluation module, using Accuracy, Specificity, Precision, and F1-score as the evaluation metrics.

## Experimental Results

### Experimental Work

In this section, the dataset and the experimental results will be explained in detail by each dataset (41). In the Animal dataset, there are 7,053 positive and 218,154 negative samples and in total there are 225,207 animal sequences. One lakh seventeen thousands one hundred and one plant sequences are taken in which 2,154 positive and 114,929 negative samples are present. One thousand four hundred six positives and 81,228 negatives and in total 82,634 human samples are given in this dataset. In Arabidopsis 231 positive samples and 28,359 negative samples in total, and there are 28,590 sequences. The virus samples are very few so it has only 1,076 in a total of which 237 are positive and 839 are negative samples (26). This is treated as a separate one and the maximum IR is only 1:400 (27). For the Animal, Plant, Human, and Arabidopsis datasets, the number of positive and negative samples for different IRs are tabulated in Table 2. For the virus, the number of positive and negative samples for different IRs is listed in Table 3. The initial target is to find the specificity and other metrics till IR up to 1:2000. Later, to improve the model it was extended to 1:5000. For the classification, here two Algorithms are used. They are ANN and Decision Tree Classifiers (10, 25, 28, 39, 40, 42–44).

**Table 2:** The no. of positive and negative samples for different Imbalance Ratios.

**IR**	**Animals**		**Plants**		**Human**		**Arabidopsis**	
	**No. of positives**	**No. of negatives**	**No. of positives**	**No. of negatives**	**No. of positives**	**No. of negatives**	**No. of positives**	**No. of negatives**
1:1	7,053	7,053	2,172	2,172	1,406	1,406	231	231
1:100	2,182	218,154	1,149	114,929	812	81,228	231	23,100
1:500	436	218,154	230	114,929	162	81,228	57	28,359
1:1000	218	218,154	115	114,929	81	81,228	28	28,359
1:1500	145	218,154	77	114,929	54	81,228	19	28,359
1:2000	109	218,154	57	114,929	41	81,228	14	28,359
1:2500	87	218,154	46	114,929	32	81,228	11	28,359
1:3000	73	218,154	38	114,929	27	81,228	9	28,359
1:3500	62	218,154	33	114,929	23	81,228	8	28,359
1:4000	55	218,154	29	114,929	20	81,228	7	28,359
1:4500	48	218,154	26	114,929	18	81,228	6	28,359
1:5000	44	218,154	23	114,929	16	81,228	6	28,359

**Table 3:** The number of positive and negative samples for different IRs for virus.

**IR**	**Virus**	
	**No. of positives**	**No. of negatives**
1:1	237	237
1:50	17	839
1:100	8	839
1:200	4	839
1:300	3	839
1:400	2	839

## Results and Discussions

**Figure 4:**
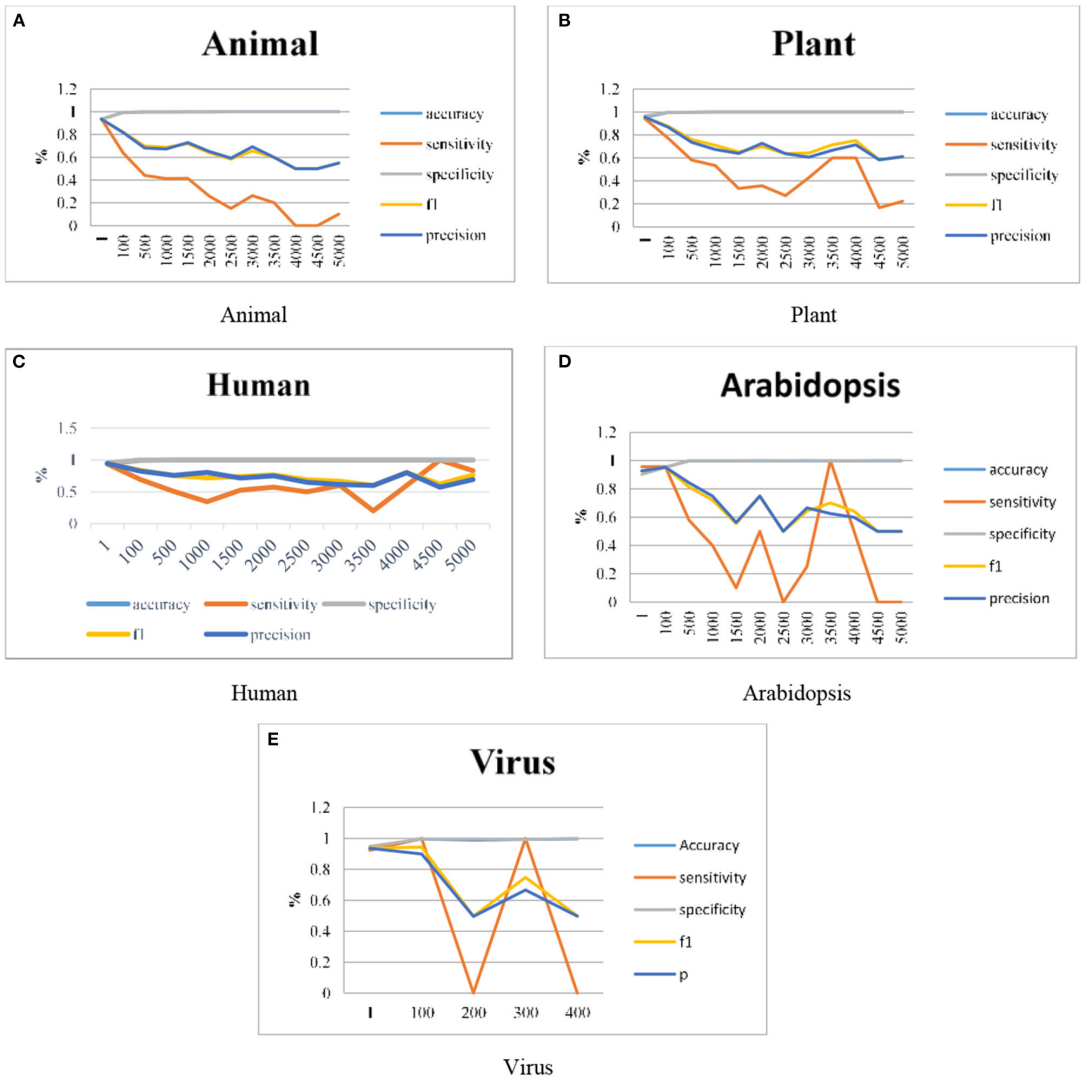
Experimental results using **(A)** Animal, **(B)** Plant, **(C)** Human, **(D)** Arabidopsis and, **(E)** Virus genome datasets.

As discussed in section Proposed Work the ANN has been integrated with the DT in our proposed work and the results are represented graphically. Graphs are plotted for clear visualization of the results. As explained in the previous sections, the model is improvised by using different datasets like Animals, Plants, Humans, Arabidopsis, and Viruses genomes. The work is extended in terms of the Imbalance Ratios also it is extended up to 1:5000. The model gives some predictions even for the 1:5000 ratio also, this can be visualized individually. Figure 4 illustrates the graphical representation of experimental results done using the proposed H-DNN framework on Animal, Plant, Human, Arabidopsis and, Virus genome datasets. The x-axis in the graph indicates the IR and the y-axis in the graph plotted illustrates the metric values. The result analysis of the proposed H-DNN Algorithm was done based on the evaluation metrics: Accuracy (Acc), Specificity (SP), Sensitivity (SE), F1-score (F1), and Precision (Prec).

First, the ANN and Decision Tree Classifier results were analyzed individually. Then their results were compared with the proposed H-DNN framework. The Experimental analysis was done by implementing the state-of-the-art ANN and DT separately and then by implementing the proposed H-DNN approach using the above-said dataset. The dataset was trained batch-wise. Table 4 illustrates the result comparison of the proposed H-DNN Algorithm with the ANN and the DT algorithms for the Animal dataset. Table 5 illustrates the result comparison of the proposed H-DNN Algorithm with the ANN and the DT algorithms for the Plant dataset.

**Table 4:** Experimental results using animal dataset.

**ANN Algorithm**						**DT Algorithm**					**Proposed H-DNN Algorithm**				
**IR**	**Acc**	**SP**	**SE**	**F1**	**Prec**	**Acc**	**SE**	**SP**	**F1**	**Prec**	**Acc**	**SE**	**SP**	**F1**	**Prec**
1	0.51	0.03	1.00	0.37	0.73	0.57	0.49	0.96	0.65	0.83	0.93	0.94	0.93	0.93	0.93
100	0.99	0.99	0.66	0.74	0.69	0.99	0.81	0.83	0.78	0.76	0.99	0.64	1.00	0.82	0.82
500	1.00	1.00	0.53	0.50	0.50	1.00	0.72	0.77	0.60	0.59	1.00	0.44	1.00	0.70	0.68
1,000	1.00	1.00	0.67	0.50	0.50	1.00	0.71	0.83	0.59	0.59	1.00	0.41	1.00	0.69	0.67
1,500	1.00	1.00	0.60	0.50	0.50	1.00	0.71	0.80	0.61	0.61	1.00	0.41	1.00	0.72	0.73
2,000	1.00	1.00	0.44	0.50	0.50	1.00	0.63	0.72	0.57	0.58	1.00	0.26	1.00	0.64	0.65
2,500	1.00	1.00	0.45	0.50	0.50	1.00	0.58	0.73	0.54	0.55	1.00	0.15	1.00	0.58	0.59
3,000	1.00	1.00	0.33	0.50	0.50	1.00	0.63	0.67	0.58	0.60	1.00	0.26	1.00	0.66	0.69
3,500	1.00	1.00	0.13	0.50	0.50	1.00	0.60	0.56	0.55	0.55	1.00	0.20	1.00	0.60	0.60
4,000	1.00	1.00	0.00	0.50	0.50	1.00	0.50	0.50	0.50	0.50	1.00	0.00	1.00	0.50	0.50
4,500	1.00	1.00	0.00	0.50	0.50	1.00	0.50	0.50	0.50	0.50	1.00	0.00	1.00	0.50	0.50
5,000	1.00	1.00	0.00	0.50	0.50	1.00	0.55	0.50	0.52	0.52	1.00	0.10	1.00	0.55	0.55

**Table 5:** Experimental results using plant dataset.

**ANN Algorithm**						**DT Algorithm**					**Proposed H-DNN Algorithm**				
**IR**	**Acc**	**SP**	**SE**	**F1**	**Prec**	**Acc**	**SE**	**SP**	**F1**	**Prec**	**Acc**	**SE**	**SP**	**F1**	**Prec**
1	0.51	1.00	0.96	0.99	0.98	0.73	0.97	0.96	0.97	0.97	0.95	0.94	0.97	0.95	0.95
100	0.99	1.00	0.55	0.76	0.49	0.99	0.88	0.77	0.82	0.68	1.00	0.77	1.00	0.87	0.87
500	1.00	1.00	0.36	0.50	0.50	1.00	0.79	0.68	0.63	0.62	1.00	0.58	1.00	0.76	0.74
1,000	1.00	1.00	0.45	0.50	0.50	1.00	0.77	0.73	0.61	0.59	1.00	0.53	1.00	0.71	0.67
1,500	1.00	1.00	0.54	0.50	0.50	1.00	0.67	0.77	0.58	0.57	1.00	0.33	1.00	0.65	0.64
2,000	1.00	1.00	0.50	0.50	0.50	1.00	0.68	0.75	0.60	0.61	1.00	0.36	1.00	0.70	0.73
2,500	1.00	1.00	0.33	0.50	0.50	1.00	0.64	0.67	0.57	0.57	1.00	0.27	1.00	0.64	0.64
3,000	1.00	1.00	0.11	0.50	0.50	1.00	0.71	0.56	0.57	0.55	1.00	0.43	1.00	0.64	0.61
3,500	1.00	1.00	0.00	0.50	0.50	1.00	0.80	0.50	0.61	0.58	1.00	0.60	1.00	0.71	0.67
4,000	1.00	1.00	0.00	0.50	0.50	1.00	0.80	0.50	0.62	0.61	1.00	0.60	1.00	0.75	0.71
4,500	1.00	1.00	0.00	0.50	0.50	1.00	0.58	0.50	0.54	0.54	1.00	0.17	1.00	0.58	0.58
5,000	1.00	1.00	0.00	0.50	0.50	1.00	0.61	0.50	0.56	0.56	1.00	0.22	1.00	0.61	0.61

Table 6 illustrates the Experimental results for the Human dataset. Table 7 illustrates the Experimental results for the Arabidopsis datasets. Table 8 illustrates the Experimental results for the Virus datasets.

**Table 6:** ANN and DT results using human dataset.

**ANN Algorithm**						**DT Algorithm**					**Proposed H-DNN Algorithm**				
**IR**	**Acc**	**SP**	**SE**	**F1**	**Prec**	**Acc**	**SE**	**SP**	**F1**	**Prec**	**Acc**	**SE**	**SE**	**F1**	**Prec**
1	0.50	1.00	0.99	0.86	0.85	0.72	0.97	0.97	0.90	0.90	0.94	0.94	0.95	0.94	0.94
100	0.99	1.00	0.79	0.50	0.50	0.99	0.85	0.89	0.67	0.66	0.99	0.69	1.00	0.83	0.83
500	1.00	1.00	0.56	0.50	0.50	1.00	0.75	0.78	0.63	0.63	1.00	0.51	1.00	0.76	0.76
1,000	1.00	1.00	0.46	0.50	0.50	1.00	0.67	0.73	0.61	0.65	1.00	0.34	1.00	0.72	0.81
1,500	1.00	1.00	0.41	0.50	0.50	1.00	0.76	0.71	0.62	0.61	1.00	0.53	1.00	0.74	0.72
2,000	1.00	1.00	0.45	0.50	0.50	1.00	0.79	0.72	0.63	0.62	1.00	0.57	1.00	0.77	0.75
2,500	1.00	1.00	0.24	0.50	0.50	1.00	0.75	0.62	0.59	0.57	1.00	0.50	1.00	0.69	0.65
3,000	1.00	1.00	0.13	0.50	0.50	1.00	0.80	0.56	0.58	0.56	1.00	0.60	1.00	0.67	0.62
3,500	1.00	1.00	0.00	0.50	0.50	1.00	0.60	0.50	0.55	0.55	1.00	0.20	1.00	0.60	0.60
4,000	1.00	1.00	0.00	0.50	0.50	1.00	0.80	0.50	0.65	0.65	1.00	0.60	1.00	0.80	0.80
4,500	1.00	1.00	0.00	0.50	0.50	1.00	1.00	0.50	0.56	0.54	1.00	1.00	1.00	0.62	0.57
5,000	1.00	1.00	0.00	0.50	0.50	1.00	0.92	0.50	0.63	0.60	1.00	0.83	1.00	0.76	0.69

**Table 7:** ANN and DT results using arabidopsis dataset.

**ANN Algorithm**						**DT Algorithm**					**Proposed H-DNN Algorithm**				
**IR**	**Acc**	**SP**	**SE**	**F1**	**Prec**	**Acc**	**SE**	**SP**	**F1**	**Prec**	**Acc**	**SE**	**SE**	**F1**	**Prec**
1	0.54	1.00	0.85	0.35	0.27	0.74	0.98	0.87	0.64	0.60	0.93	0.96	0.90	0.93	0.93
100	0.54	1.00	0.75	0.35	0.27	0.75	0.98	0.85	0.65	0.61	0.95	0.96	0.95	0.95	0.95
500	1.00	1.00	0.12	0.50	0.50	1.00	0.79	0.56	0.66	0.67	1.00	0.58	1.00	0.81	0.84
1,000	1.00	1.00	0.25	0.50	0.50	1.00	0.70	0.63	0.61	0.62	1.00	0.40	1.00	0.72	0.75
1,500	1.00	1.00	0.55	0.50	0.50	1.00	0.55	0.77	0.53	0.53	1.00	0.10	1.00	0.56	0.56
2,000	1.00	1.00	0.24	0.50	0.50	1.00	0.75	0.62	0.62	0.62	1.00	0.50	1.00	0.75	0.75
2,500	1.00	1.00	0.00	0.50	0.50	1.00	0.50	0.50	0.50	0.50	1.00	0.00	1.00	0.50	0.50
3,000	1.00	1.00	0.00	0.50	0.50	1.00	0.63	0.50	0.57	0.58	1.00	0.25	1.00	0.64	0.67
3,500	1.00	1.00	0.00	0.50	0.50	1.00	1.00	0.50	0.60	0.56	1.00	1.00	1.00	0.70	0.63
4,000	1.00	1.00	0.00	0.50	0.50	1.00	0.75	0.50	0.57	0.55	1.00	0.50	1.00	0.64	0.60
4,500	1.00	1.00	0.00	0.50	0.50	1.00	0.50	0.50	0.50	0.50	1.00	0.00	1.00	0.50	0.50
5,000	1.00	1.00	0.00	0.50	0.50	1.00	0.50	0.50	0.50	0.50	1.00	0.00	1.00	0.50	0.50

**Table 8:** ANN and DT results using virus dataset.

**ANN Algorithm**						**DT Algorithm**					**Proposed H-DNN Algorithm**				
**IR**	**Acc**	**SP**	**SE**	**F1**	**Prec**	**Acc**	**SE**	**SP**	**F1**	**Prec**	**Acc**	**SE**	**SP**	**F1**	**Prec**
1	0.52	0.48	0.99	0.52	0.52	0.86	0.94	0.92	0.86	0.92	0.94	0.92	0.95	0.94	0.94
100	0.99	1.00	0.32	0.50	0.49	0.99	1.00	0.92	0.90	0.93	1.00	1.00	1.00	0.94	0.90
200	0.99	1.00	0.16	0.50	0.50	0.99	1.00	0.96	0.48	0.44	0.99	0.00	1.00	0.50	0.50
300	1.00	1.00	0.00	0.50	0.50	0.99	0.00	0.98	0.76	0.65	0.99	1.00	0.99	0.75	0.67
400	1.00	1.00	0.00	0.50	0.50	1.00	0.00	1.00	0.41	0.49	1.00	0.00	1.00	0.50	0.50

By analyzing the results, we observed that the ANN gives better results and gives a good True Positive Ratio (TPR) with 2 epochs and up to IR 1:2000. If the model is trained further for more epochs, then we are getting an over-fitted model. The time required is also in milliseconds which is very optimal whereas the existing system takes hours to train one epoch. Furthermore, when the model needs to be trained with IR up to 1:5000 Decision Tree Classifier gives better results with good TPR. But when we integrate both the DT and the ANN we get a better result for almost all the IRs up to 1:5000 as compared to the performance gained by DT and ANN separately.

## Conclusion

The production of imbalanced data in large quantities in Bioinformatics provides a great scope for work in the AI world. These kinds of problems require modern solutions for which many questions will arise that have to be justified and have to satisfy all the complex conditions too. One such problem is the classification of these miRNA sequences which are very few in genome data and are similar to other RNA structures. The miRNA seems to help detect and diagnose cancer disease. Hence classifying this miRNA is a big challenge that has to be faced. So, a novel solution is required with minimal complexity that provides better results in terms of the TPR, time, and accurate classification of miRNA from the imbalanced genome dataset. In this paper, we have proposed a Hybrid Neural Network model in which we have integrated Deep ANN and Deep Decision Tree Classifiers. The Deep ANN gives better results when compared with the existing result and good TPR with 2 epochs and up to IR 1:2000. Furthermore, when the model needs to be trained with IR up to 1:5000, Deep Decision Tree Classifier gives better results with good TPR than ANN. When we integrate both Deep ANN and Deep DT we get better performance as compared to both taken individually. In our future work, we would like to further extend by increasing the IR and reducing the time of convergence. We would also like to check the performance of the proposed model on other large genome datasets. The main aim is that the model should not follow any sort of sampling techniques and predict the miRNAs with good TPR.

## Data Availability Statement

The original contributions presented in the study are included in the article/supplementary material, further inquiries can be directed to the corresponding author/s.

## Author Contributions

ER, SR, and RE contributed in problem formulation, implementation and results. KK, SP, SV, VV, and AM contributed in methodology identification and techniques to project results. All authors contributed to the article and approved the submitted version.

## Conflict of Interest

The authors declare that the research was conducted in the absence of any commercial or financial relationships that could be construed as a potential conflict of interest.

## Publisher's Note

All claims expressed in this article are solely those of the authors and do not necessarily represent those of their affiliated organizations, or those of the publisher, the editors and the reviewers. Any product that may be evaluated in this article, or claim that may be made by its manufacturer, is not guaranteed or endorsed by the publisher.
